# Emerging Trends in Noninvasive Insulin Delivery

**DOI:** 10.1155/2014/378048

**Published:** 2014-05-14

**Authors:** Arun Verma, Nitin Kumar, Rishabha Malviya, Pramod Kumar Sharma

**Affiliations:** Department of Pharmacy, School of Medical & Allied Sciences, Galgotias University, Yamuna Expressway, Greater Noida, Uttar Pradesh, India

## Abstract

This paper deals with various aspects of oral insulin delivery system. Insulin is used for the treatment of diabetes mellitus, which is characterized by the elevated glucose level (above the normal range) in the blood stream, that is, hyperglycemia. Oral route of administration of any drug is the most convenient route. Development of oral insulin is still under research. Oral insulin will cause the avoidance of pain during the injection (in subcutaneous administration), anxiety due to needle, and infections which can be developed. Different types of enzyme inhibitors, like sodium cholate, camostat, mesilate, bacitracin, leupeptin, and so forth, have been used to prevent insulin from enzymatic degradation. Subcutaneous route has been used for administration of insulin, but pain and itching at the site of administration can occur. That is why various alternative routes of insulin administration like oral route are under investigation. In this paper authors summarized advancement in insulin delivery with their formulation aspects.

## 1. Introduction

Diabetes mellitus is a metabolic disorder which is characterized by the elevated glucose level (above the normal range) in the blood stream that is hyperglycemia. Millions of people in the world are affected by diabetes. It has been studied that approximately 7% of the people in US are affected by diabetes. Insulin is a polypeptide hormone required to be taken in patients suffering from diabetes mellitus. It is an anabolic hormone. Due to insulin glucose uptake by the cells of different organs (like muscle, liver, fat cells, etc.) from the blood can be possible. It stores the glucose in muscle and liver as glycogen. In the absence of insulin, cells of different organs cannot take the glucose through blood. In this condition fat is used as an energy source. Any imbalance in the level of insulin causes diabetes mellitus. It is produced by islets of Langerhans (clusters of cells that are embedded in exocrine portion of pancreas) [1].

Insulin which is very close to the human insulin is porcine insulin. Insulin controls the energy metabolism with the help of epinephrine and norepinephrine. It is the hormone which plays a very important role in use of fuels by tissues. Insulin is stored in cytosol and released by exocytosis. Metabolism of insulin takes place by enzyme* Insulinase*. Half-life of insulin is approximately 6 minutes. Insulin secretion is increased by an increase in glucose, amino acids, and gastrointestinal hormones. Release of insulin is decreased when there is a scarcity of dietary fuels, during stress (fever and infection). Mechanism of action of insulin is it binds to specific, high affinity receptors in cell membranes of tissues (liver, muscle, adipose) [2]. Therapeutic proteins such as insulin (due to their specificity) are drug of choice for treating diseases [3]. This paper is presented to review the various routes of administration of insulin.

## 2. Oral Delivery of Insulin

Oral route of administration of any drug is the most convenient route among others. Development of oral insulin is still under research. Administration of insulin through oral route causes the avoidance of pain during the injection (in subcutaneous administration), anxiety due to needle, and infections which can be developed. Oral insulin delivery is beneficial due to its direct delivery to liver. Dynamics of subcutaneous delivery of insulin is not similar to the normal endogenous insulin release [4–12]. A very low permeability of insulin occurs across epithelium of intestine because of two reasons, first is because of its high molecular weight and the fact that it is degraded very rapidly by enzyme* Insulinase*. Oral bioavailability of proteins like insulin is below 1%. So there is a target to improve it to about 30%–50%. Metabolism or degradation of insulin takes place by certain enzymes like pepsin, trypsin, chymotrypsin, so the insulin is degraded by acidic environment of stomach. Insulin degrading enzymes (IDEs) which are also known as cytosolic enzymes degrade the insulin [13, 14]. To overcome these problems regarding the oral insulin delivery three approaches could be possible. By changing the following:

- physicochemical properties of the insulin, for example, lipophilicity;
- crosslinking with macromolecules;
- use of carrier systems.

Different types of enzyme inhibitors, like sodium cholate, camostat mesilate, bacitracin leupeptin, FK-448, and so forth, have been used to prevent insulin metabolism by which increased amount of insulin is available for absorption [15–19].

Different types of penetration enhancers can be used to increase the absorption of insulin* via* intestinal epithelium. Excipient such as surfactants (ethylenediaminetetraacetic acid) has been used to increase the absorption of protein drugs like insulin. In some study intestinal permeation enhancers like mucoadhesive polymers have been used for mucosal delivery of insulin [20]. Different types of carriers like liposomes, microspheres, and nanoparticles have been used for delivery of protein. Insulin was incorporated in these carriers to prevent its gastrointestinal degradation. By this approach the absorption and bioavailability of insulin can be enhanced [21]. Hydrogels have also been used as carrier that protects insulin degradation in acidic environment of stomach and allows safe transportation in the intestine [22].

Emispheres are other carrier systems which have been used in the oral delivery of insulin. They are proteinoids in nature. Condensation of different types of polymers gives these types of delivery systems. Emispheres are capable of binding with transporters present on cell membrane. By these transporters the emispheres which are carrying the prodrug can cross the cell membrane. Where the complex of emisphere and the transporter dissociates and drug returns to its biological active form [23].

Nanoparticles are also used as insulin carriers and it is an extensively newer approach to deliver insulin. Amount of insulin and polymer plays an important role in the therapeutic efficacy of insulin. In* in vitro* studies it was observed that nanoparticles protect the insulin through enzymatic degradation. Polymers mainly used in formulation of nanoparticles are polyalkylcyanoacrylate, polymethacrylic acid, and polylactic-co-glycolic acids (PLGA). Polymers like chitosan, alginate, gelatin, albumin, lectin, and so forth are also used which are found naturally. Among these chitosan has a good permeation property. In a diabetic rat model nanoparticle with chitosan significantly reduces the blood glucose level [24].

Nanoparticles or microparticles of poly (D,L-lactide-co-glycolide)- (PLAGA-) based polymers are biodegradable in nature and have been widely used as carriers for controlled drug delivery of molecules which are susceptible to degrade like peptides, proteins, antigens, hormones, and so forth. Methods of preparation of these devices that have been used are emulsions or double emulsion technique, solvent evaporation, or spray drying. Some factors should be optimized during preparation of these devices like release rates, encapsulation efficiency, and so forth, to improve their therapeutic efficacy [25].

Insulin delivery from oral delivery devices is a better approach to overcome the frequent administration of subcutaneous injections of insulin. Polymeric devices have been widely used for oral insulin delivery through hydrogels, nanoparticles, or microparticles.

Interpolymer complexes of the grafted copolymers also have been a good approach for the oral insulin drug delivery [26]. Polymeric nanoparticles having biodegradable property are good candidates for oral insulin delivery [27].

Surface active agents like polysorbates and sodium dodecyl sulphate stabilize the insulin formulation. Absorption of vitamin B 12 may be helpful in passive cotransport of insulin. Hexyl insulin monoconjugate 2 (HIM 2) is a recent development in the oral insulin therapy; it is well tolerated and relatively safer [28].

Aboubakar et al. investigated that poly(isobutylcyanoacrylate) nanocapsules prevent enzymatic degradation of insulin. Study reveals the fact that poly(isobutylcyanoacrylate) nanocapsules play an important role in absorption of insulin and when they are administered orally, they provide active form of insulin [29].

Prusty et al. prepared the nanoparticles incorporated with insulin by the complex coacervation method. They have been studied antidiabetic activity of orally administered insulin in rats. These nanoparticles have been evaluated for entrapment efficiency, particle size, and* in vitro* release studies,* in vivo* pharmacological studies, pharmacokinetic evaluation, and biochemical parameters. Insulin present in serum has also been determined by the use of human insulin ELISA kit. Biochemical parameters like creatinine and protein levels in the serum were estimated by spectrophotometry. Particle size of nanoparticles loaded with insulin was observed as 551.67 nm ± 45.5 and it was determined by scanning electron microscopy (SEM). Loading efficiency of nanoparticles containing insulin was found highest for 50 IU/mL insulin loaded nanoparticles and lowest for 51 IU/mL insulin loaded nanoparticles. Korsmeyer's equation was used in* in-vitro* studies for determination of drug release pattern. The release of kinetics was found to be 0.18.* In vivo* antidiabetic studies showed the significant reduction of serum glucose level. This serum glucose level was sustained for longer period of time. Percentage pharmacological bioavailability of nanoparticles containing 10 IU/Kg was found to be 43.60. They also determined the body weight of the animals. It has been observed that the reduced weights of the animals were increased after the administration of nanoparticles containing insulin. Oral bioavailability was found to be less than 50% but the pharmacological effects of insulin have been noticed for a longer period of time with the nanoparticle as compared to parenteral insulin administration. They concluded that 10 IU/Kg of orally administered insulin given to diabetic rats which is incorporated in nanoparticles shows a maximum change in glucose level of at a time period of 5 hours [30].

Herrath et al. investigated that insulin administrated by oral route prevents diabetes induced by virus in transgenic mouse model. It has been proposed that immunotherapy using self-antigens can be used to treat autoimmune diseases. As aberrant immune responses and probably viruses are involved in pathogenesis of insulin dependent diabetes mellitus they studied oral insulin as self-antigen in transgenic mouse model to treat virus-induced insulin dependent diabetes mellitus. Rat insulin promoter-GP34-20 (RIP-GP34-20) transgenic mice were used for rapid onset IDDM (insulin dependent diabetes mellitus) and RIP-NP 25-3 mice were used for slow onset IDDM. LCMV ARM (clone 53b) and vaccinia virus recombinants that express LCMV-GP (lymphocytic choriomeningitis virus-GP) aa1-398(vv/GP) has been used to induce IDDM. Blood glucose has been checked using ACCUCHECK II. They noted that giving a self-protein (oral insulin) is effective when used either before or after autoimmune trigger (specific virus). They concluded that orally administered insulin prevents diabetes in transgenic mouse [31].

Choudhari et al. formulated and evaluated insulin incorporated in liposomes. Furthermore they evaluated effect of formulation after oral administration. They concluded that the effect of this system was as same as the 1 unit of insulin which is administered by subcutaneous route [32].

Najafzadeh et al. evaluated efficacy of formulation of insulin having polar and nonpolar ingredients which has been administered by oral route. They concluded that novel excipients used in formulation prevent the degradation of insulin from gastric enzymes. This formulation significantly reduces the concentration of glucose in blood plasma in healthy and diabetic rats [33].

Elsayed et al. investigated possibility of administration of insulin by oral route by using nanocapsulation and use of vehicle having oily phase. They concluded that orally administered nanoparticles increase the effectiveness of insulin administered by oral route [34].

Mundargi et al. investigated that copolymeric hydrogels which are pH sensitive synthesized from N-vinyl-caprolactam and methacrylic acid monomers through free radical polymerization result in 52% encapsulation efficiency. They observed that in* in vitro* studies insulin loaded microparticles showed no release of insulin for at least 2 hours in stomach and 100% insulin was released in intestinal media for 6 hrs. These hydrogels have been evaluated by Fourier transform infrared spectroscopy, differential scanning calorimeter, thermogravimetry, and nuclear magnetic resonance for conformation of copolymer formation. Scanning electron microscopy has been used to evaluate the morphology of hydrogel microparticles. In* in vivo* studies diabetic rats, those induced by alloxan, showed 50% biological inhibition and 44% inhibition by glucose tolerance test [35].

Mundargi et al. prepared pH sensitive microspheres from Eudragit 100 and Eudragit RS 100. They studied their* in vitro* and* in vivo* release data to evaluate them. These microspheres have been prepared by double emulsion solvent evaporation method for oral insulin delivery. They have been used Eudragit L 100 for preclinical studies. The insulin has been entrapped in microspheres and various* in vitro* tests have been performed on insulin loaded microspheres in acidic (pH 1.2) media but maximum release was observed in buffer media (pH 7.4) from 4 to 6 hrs. Size of microspheres has been observed from 1 to 40 *μ*m. Structural configuration of insulin was observed in circular dichroism spectra even after its release in buffer media (pH 7.4).* In vivo* release studies showed that diabetic induced rat models showed a maximum inhibition of 86% which indicates that insulin was absorbed through intestine [36].

## 3. Buccal Route of Insulin Administration

The lining of inner cheek is called buccal mucosa and when the insulin is absorbed by this buccal mucosa and reached to systemic circulation by placing buccal formulation inside the mouth, that is, between upper gingiva and cheek, and then it is called as buccal insulin. Factors which influence the absorption potential are molecular weight, hydrophilicity, conformation stereo specificity, solubility, electrostatic charge, and partition coefficient of proteins and peptides. In this type of delivery the insulin is absorbed in mouth and throat through applying a device which delivers the insulin in spray form [37–47].

In the buccal mucosa the blood supply in reticulated veins is very high, so absorption will be also high and this route prevents hepatic first pass metabolism. Delivery by inhaler containing high pressure droplets of insulin to the back of the throat is beneficial. Due to low permeability of buccal mucosa more puffs are required for optimum drug delivery [48].

Bioadhesive formulations are the best method for insulin delivery through buccal mucosa, for example, gels, films, nanoparticles, and sponges. Time of application of dosage form affects the absorption profile of plasma insulin. Maximum 12% pharmacological activity has been noted when insulin in buccal mucosa is administered with absorption enhancers [49].

For mucosal delivery in controlled manner pluronic F-127 gel has been used, as it has weak immunogenic property, low toxicity, bioadhesive nature. At 20% or more concentrations in aqueous solutions, it behaves as a reversible thermal gelatin [50].

Transferosomes are modified liposomes get deform for easy penetration through the pores and release gradually. Water soluble drugs are delivered transdermaly by transfersomes. These can also be used for buccal administration of insulin [51].

Pelleted nanoparticles have been used for buccal administration of insulin as they have 3D (three-dimensional) structural conformity and coherence and facilitate buccal application and adherence [52, 53]. Buccoadhesive tablets using compression of powder mixes (carbopol 934, hydroxylpropyl methylcellulose, and few absorption promoters) have been used for insulin buccal delivery, but patches are considered to be better than tablets as these are thin and flexible. It also consists of drug reservoir, impermeable backings, bioadhesive surface, and so forth. Sodium deoxycholate is the best for enhancing permeation so patches are among good approaches for insulin delivery. Even films are considered to be better than adhesive tablets but they should be strong enough to avoid breakage in the mouth. Bilayered system of insulin delivery is sponges. These sponges are also mucoadhesive (chitosan layer and impermeable ethyl cellulose layer), porous, and flexible. By modifying formulation variables, for example, type of chitosan salt insulin content molecular weight, we can control* in vitro* release properties from sponges. For insulin buccal spray soybean lecithin and propanediol are used. Studies show that intracellular and paracellular, both routes, have been used in the passage of FITC (fluorescein isothiocyanate) insulin through buccal mucosa. Only marketed buccal insulin formulation is Ora-lyn. It is a liquid formulation and is used with a propellant. Rapidmist technology is used for insulin delivery which sprays insulin directly in the mouth towards throat and it delivers aerosols of high velocity so that it is absorbed rapidly by buccal mucosa into the systemic circulation [54–59].

Bhumkar et al. investigated a novel method to synthesize gold nanoparticles, which increases the penetration of insulin through mucosal layer. Chitosan (a natural cationic polysaccharide obtained from chitin) has been used for the synthesis of gold nanoparticles. Here chitosan acts as a reducing agent and penetration enhancer. In this preparation bovine insulin, chloroauric acid, and alloxan have been used. Chitosan having concentrations 0.05, 0.01, 0.1, 0.2 has been used for the reduction of chloroauric acid to produce gold nanoparticles. Insulin has been loaded onto gold nanoparticles and percentage loading efficiency has been calculated. They have been using UV spectroscopy (ultraviolet spectroscopy) to observe the change in surface plasmon resonance of gold nanoparticles. Evaluation parameters like transmission electron microscopy, particle size analysis, viscosity measurements,* in vitro* dissolution studies, zeta potential measurements, fluorescence spectroscopy measurements,* in vivo* studies, and inductively coupled plasma (ICP) measurements have been performed. ICP measurements were used to determine the gold concentration in the blood serum after administration of different formulations of gold nanoparticles to rats which were diabetic. They concluded that prepared gold nanoparticles containing insulin controlled postprandial increased glucose level and insulin loaded chitosan reduced gold nanoparticles showed increased pharmacodynamic activity when they were administered by oral route and nasal route. A significant reduction in serum glucose level had been observed [60].

Prasanth et al. developed a buccal insulin drug delivery system through using locust bean gum to increase the permeability and effectiveness of insulin. Locust bean gum acts as a mucoadhesive polymer. Therapeutically locust bean gum has been acting as adsorbent, demulscent, and stabilizer. They have used polyethylene glycol dimethyl ether 500 which act as permeation enhancer. Direct compression method has been used to prepare buccal tablets of insulin. Various evaluation parameters have been performed on prepared tablets like* in vitro* bioadhesion study,* in vitro* drug release, drug permeation, and* in vivo* study. They concluded that locust bean gum, DME500 (dimethyl ether), and PEG (polyethylene glycol) were suitable candidates for buccal insulin drug delivery [61].

Portero et al. developed a new device for peptide administration through buccal route. This device is composed of chitosan layer (mucoadhesive in nature) in which peptide drug and ethyl cellulose were dispersed, where ethyl cellulose acts as protective impermeable layer. This system has been responsible for unidirectional release of drug through mucosal layer. This avoided the loss of drug due to salivary secretion. Release of insulin has been facilitating by changing some variables like type of chitosan salt, pH of chitosan solution, and dose of insulin. Release of insulin has been affected by diffusion through polymer matrix. Studies show that chitosan sponges have a good affinity to the mucosal surface and it is related to the different properties of the different salt of the chitosan [56].

## 4. Transdermal Approach of Insulin Delivery

Characteristics of transdermal insulin drug delivery system are given below:

- It gives passive delivery of insulin.
- patch, cream, and spray forms can be used;
- it requires a day to diffuse through skin and to have systemic effect.

Drawback of transdermal drug delivery is that insulin molecules are large enough to penetrate the skin at therapeutically useful rates. So nowadays microneedles are being fabricated with different sizes, shapes, and so forth to increase transdermal delivery. Various* in vivo* and* in vitro* studies have shown better results with solid microneedles. Needle arrays increase the transport by diffusion and iontophoresis and as drug carriers.

## 5. Sonophoresis

Application of ultrasound at a low frequency also increases skin permeability [62].

Chen et al. investigated the nanovesicles which can increase permeability of insulin through iontophoresis. The results show that nanovesicles increases permeation with the help of microneedles and iontophoresis [63].

Chien et al. developed a noninvasive drug delivery technique for controlled drug delivery of peptides to the various organs and tissues. They established a relationship between the pharmacodynamics and pharmacodynamics and also analyzed iontophoresis-facilitated transdermal transport technique for peptides [64].

Martanto et al. prepared solid microneedles for administration of insulin through transdermal route to decrease plasma glucose level. They concluded that arrays of microneedles have the ability to increase permeability of skin to insulin [65].

Malakar et al. developed microemulsions containing insulin for transdermal drug delivery. They concluded that prepared microemulsions increase the permeation of insulin through transdermal route [66].

Guo et al. evaluate the prepared lecithin vesicles possessing insulin and estimate the effect of these vesicles on transdermal drug delivery system. They concluded that these vesicles may be a good carrier for the delivery of insulin through transdermal route [67].

Park et al. determined the usefulness of lightweight cymbal transducer array for delivery of insulin through transdermal route. They concluded that cymbal array was a useful system for delivery of insulin through transdermal route [68].

## 6. Inhalation Approach of Insulin Delivery

This route of administration has relatively faster onset of action than subcutaneous route and with less degradation by proteolytic enzymes. Inhaled insulin has longer glucose lowering activity. Absorption of inhaled insulin occurs through mucosal surface of lungs (alveoli, approximate surface area 100 meter square). Exubera, insulin human (originated from ribosomal DNA) inhalation powder has also been approved by FDA (food and drug administration) for children more than 6 years and adult patient.

- For obese patients: it has been found that inhaled insulin absorption is independent of body mass index.
- For smokers: more rapid onset of action has been seen.
- Greater maximum effect has been seen.
- Greater total glucose lowering effect has been seen.
- In asthmatic patients: it has been found that in absence of bronchodilator, absorption was 20% less than patients with nonasthmatic patients.
- COPD (chronic obstructive pulmonary disease): systemic insulin concentrations have been twofold more.
- Bioavailability of inhaled insulin does not increase by holding breath at the end of inspiration.
- Dose is given within 10 minutes before meal.
- Exubera: pregnancy risk category C.
- Subcutaneous insulin: pregnancy risk category B [69–74].

Cefalu et al. found out the efficacy and safety of insulin which is delivered by pulmonary route. In their study they included 26 participants, 16 men and 10 women with average age 51.1 year and average duration of diabetes was 11.2 years. Patient randomly received inhaled insulin (14.6 ± 5.1 mg) and ultralente insulin (35.7 ± 18.4 U) daily in comparison to 19 units of regular insulin and 51 units of long-acting insulin at the baseline. Glycemic control improved significantly with inhaled insulin. HbA_1c_ (glycosylated hemoglobin) levels reduced from 0.0867 ± 0.0144 (8.67% ± 1.44%) at the baseline to 0.0796 ± 0.0137 (7.96% ± 1.37%). They also did not detect any change in lung parameters like volumes, spirometry results, diffusion capacity, and oxygen saturation. 69% of patients experienced mild to moderate hypoglycemia. No severe event was noted. They concluded that insulin delivered by pulmonary route shows a good glycemic control and it shows no adverse pulmonary effects [75].

Setter et al. studied the clinical pharmacokinetics, tolerability, adverse events, dosage and administration, comparative efficacy, and cost of inhaled dry powder insulin. They concluded that it provides a better control of HbA_1c_ [76].

Leal et al. compared the role of rapid-acting insulin analogues and inhaled insulin in the treatment of diabetes. They concluded that use of rapid-acting insulin analogues gives a good control of higher glucose level. They also concluded that it also reduces the cardiovascular risk conditions [77].

Skyler et al. studied the safety of pulmonary route when exubera, that is, inhaled human insulin is administered by the young patients with insulin dependent diabetes mellitus. They concluded that exubera had no adverse effect on lung function [78].

Reger et al. studied the effect of intranasal insulin in memory-impaired older adults. On the basis of studies it has been proven that insulin administration through nasal route modulates the verbal memory and plasma *β*-amyloid in memory impaired older adults. This modulation was dose dependent. This is beneficial for Alzheimer patients. They studied the cognitive dose response curves for insulin administration through nasal route. They determined the difference in effect of insulin between participants with *ε*4 and without *ε*4 allele. Five treatment conditions of intranasal insulin, that is, 10, 20, 40, or 60 IU, have been applied to 33 memory-impaired adults suffering from Alzheimer's disease or amnestic mild cognitive impairment and 59 adults without Alzheimer's disease that were normal adults. After insulin administration through nasal route, cognition was tested. Glucose level in plasma was unaffected after treatment. This has concluded that nasal insulin administration modulates the verbal memory in memory impaired *ε*4-adults at 20 IU of dose. It has been observed that a relative decrease in verbal memory showed in *ε*4+ adults. Nasal insulin also facilitates the plasma *β*-amyloid in memory-impaired patients. They have concluded that subjects with different genetic risks for Alzheimer's disease showed different responses to the nasal insulin administration and the responses were dose dependent [79].

A device named as Afrezza has been used for insulin administration through inhalational route. This device composed of Technosphere insulin inhalational powder with a breath activated inhaler. For the treatment of type 1 and type 2 diabetes mellitus cartridges of Technosphere insulin (TI) should be used with an inhaler before taking meal. Postprandial glucose and glycated hemoglobin significantly reduce in type 2 diabetes patients after administration of Technosphere insulin [80].

Some limitations of insulin delivery through pulmonary route are its expensiveness and that the bioavailability of insulin from pulmonary route is about 9%–22% of that of subcutaneous route, absorption of insulin is variable due to smoking, respiratory infections, and some age related factors, and insulin is required in large quantity as compared to other routes [81]. 

## 7. Patents

Sharma et al. invented a method for the synthesis of microcapsules containing insulin which were administered orally. In this method they used an emulsified solution of chitosan in a vegetable oil [82].

Ecanow invented the method for preparation of oral dosage form of insulin. Their idea is based on a biphasic liquid aqueous system containing insulin. They also produce oral dosage form of insulin administered orally having sustained release characteristics [83].

Burnside et al. invented an emulsion having insulin encapsulated in a carrier which is a good candidate for oral delivery of insulin. They use an emulsion having hydrophobic continuous phase which is dispersed in an aqueous phase (discontinuous phase) [84].

Arbit et al. invented that oral administration of insulin decreases the possibilities of vascular diseases which arises due to repeatedly administered insulin. They also invented that insulin administration through oral route increases the absorption of insulin through gastrointestinal mucosa [85].

Qian et al. invented enteric coated nanoparticles having insulin. Purpose of this invention was to prevent the insulin from acidic and enzymatic degradation. They use cationic nanoparticle comprising a polycationic, muocoadhesive polymer and a biodegradable polymer. They concluded that cationic nanoparticles facilitate the permeability of insulin [86].

Arbit et al. invented a method for the treatment of diabetes due to impaired tolerance of glucose. This method gives an improved glucose control and does not have any risk of hypoglycemia, hyperinsulinemia and no risk of weight gain [87].

Gold Berg et al. invented a method for treatment of impaired tolerance of glucose or diabetes mellitus which is in early stage. They designed a dosage form comprising insulin which is effective at night time or bed time [88].

Sabetsky et al. invented a method which lowers glucose level in mammals using crystallized dextran microparticles. For controlled release of insulin, composition of formulation can be either one phase or multiphase [89].

Zhang et al. invented an oral formulation containing insulin which gives specific drug delivery in colon [90].

Gonda et al. invented a device which measures the inhaled volume and also adjusts the applied force to container having formulation to deliver the drug from it. This device also has the facility to adjust the amount of force which is applied to the container having formulation to expel the drug. Amount of the drug which is expelled depends on the force applied [91].

Gonda et al. invented a method and a device which eliminated the need of injected insulin. When aerosolized monomeric insulin is delivered by inhalational route the rate of absorption of insulin is increased. This device has an electronic sensor by which we can measure the inspiratory flow [92].

Steiner et al. found invented that combination of a chelator and an acidifier gives more rapid absorption without chelator and an acidifier. This formulation is suitable for injectable route of administration or the pulmonary or oral route of administration but subcutaneous route is preferred [93].

Schmitke et al. invented a method for delivery of insulin by pulmonary route. The insulin delivered in this fashion gives a rapid release. They invented a formulation having phospholipids, drug, and excipient. They formulate this formulation in a powdered form [94].

Brush et al. invented dry powdered insulin for diabetes through pulmonary route which gives rapid release. They invented a formulation having phospholipids biologically active agent and excipient. They formulate improved powdered inhaled insulin [95].

Backstorm et al. invented a formulation of inhaled insulin which increases the absorption of insulin in lower respiratory tract. Powdered formulation is used for this purpose [96].

Patton et al. invented a dry powder formulation of insulin which provides rapid absorption through alveoli [97].

Gonda et al. invented a method and a device which eliminate the need of injected insulin. When aerosolized monomeric insulin was delivered by inhalational route the rate of absorption of insulin was increased. This device has an electronic sensor by which we can measure the inspiratory flow [98].

Backstorm et al. invented a formulation having a mixture of therapeutically and pharmaceutically active compounds which facilitate the absorption of polypeptides like insulin in the lower respiratory tract. Mixture is prepared in the dry powdered form [99].

Gonda et al. invented a method and a device which eliminated the need of injected insulin. When aerosolized monomeric insulin is delivered by inhalational route the rate of absorption of insulin is increased. This device has an electronic sensor by which we can measure the inspiratory flow. They conclude that the rate of lowering the concentration of glucose in blood is increased by the use of monomeric insulin [100].

Modi et al. invented a formulation having a macromolecular pharmaceutical agent in a micellar form. They also invented the method for making and using composition. They were preferred buccal route for the administration of formulation [101].

Modi et al. invented a formulation having a proteinic pharmaceutical agent and one absorption enhancing compound among alkali metal salicylate, edetate, C8–C22 alkyl sulfate, and so forth. They also invented method for insulin administration through buccal route [102].

## 8. Conclusion

Protein and peptide can be degraded in gastrointestinal tract when administered through oral route. Thus to avoid degradation, they are generally administered through parenteral route. It can be concluded from literature survey that insulin can be safely delivered into systemic circulation through buccal route, transdermal route, and nasal route.
